# When DNA Polymerases Multitask: Functions Beyond Nucleotidyl Transfer

**DOI:** 10.3389/fmolb.2021.815845

**Published:** 2022-01-07

**Authors:** Denisse Carvajal-Maldonado, Lea Drogalis Beckham, Richard D. Wood, Sylvie Doublié

**Affiliations:** ^1^ Department of Epigenetics and Molecular Carcinogenesis, The University of Texas MD Anderson Center, Houston, TX, United States; ^2^ Department of Microbiology and Molecular Genetics, University of Vermont, Burlington, VT, United States

**Keywords:** DNA polymerases, nucleotidyl transfer, DNA repair, nuclease activity, lyase activity, proofreading

## Abstract

DNA polymerases catalyze nucleotidyl transfer, the central reaction in synthesis of DNA polynucleotide chains. They function not only in DNA replication, but also in diverse aspects of DNA repair and recombination. Some DNA polymerases can perform translesion DNA synthesis, facilitating damage tolerance and leading to mutagenesis. In addition to these functions, many DNA polymerases conduct biochemically distinct reactions. This review presents examples of DNA polymerases that carry out nuclease (3ʹ—5′ exonuclease, 5′ nuclease, or end-trimming nuclease) or lyase (5′ dRP lyase) extracurricular activities. The discussion underscores how DNA polymerases have a remarkable ability to manipulate DNA strands, sometimes involving relatively large intramolecular movement.

## Introduction

**FIGURE 1:**
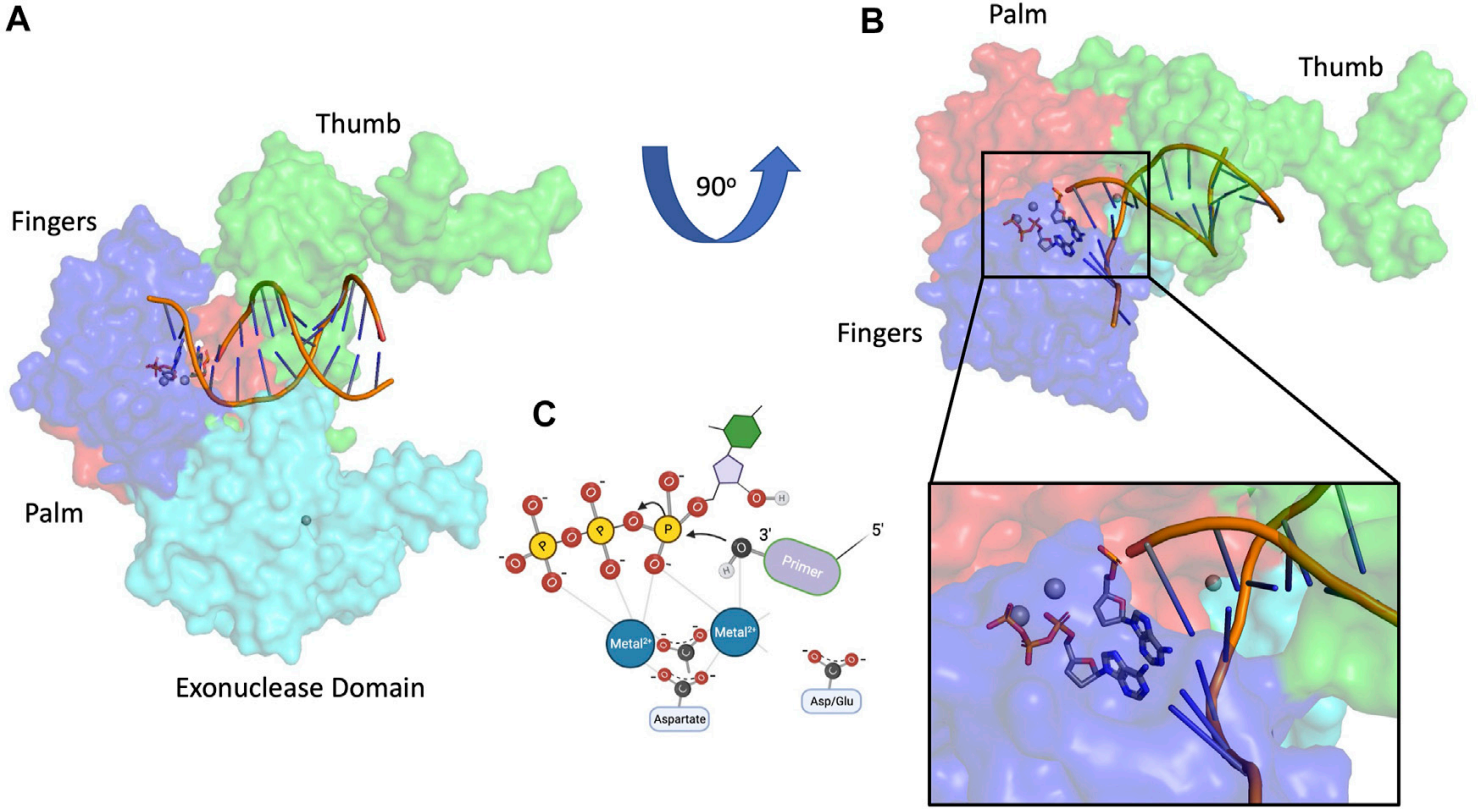
Canonical DNA polymerase nucleotidyl transfer activity. **(A)** T7 DNA polymerase structure 1SKR (Li et al., 2004), **(B)** T7 DNA polymerase rotated 90^o^ with magnified inset, **(C)** two-metal DNA polymerase mechanism. Subdomain color scheme: fingers (blue), palm (red), thumb (green), 3′-5′ exonuclease (cyan).

**FIGURE 2:**
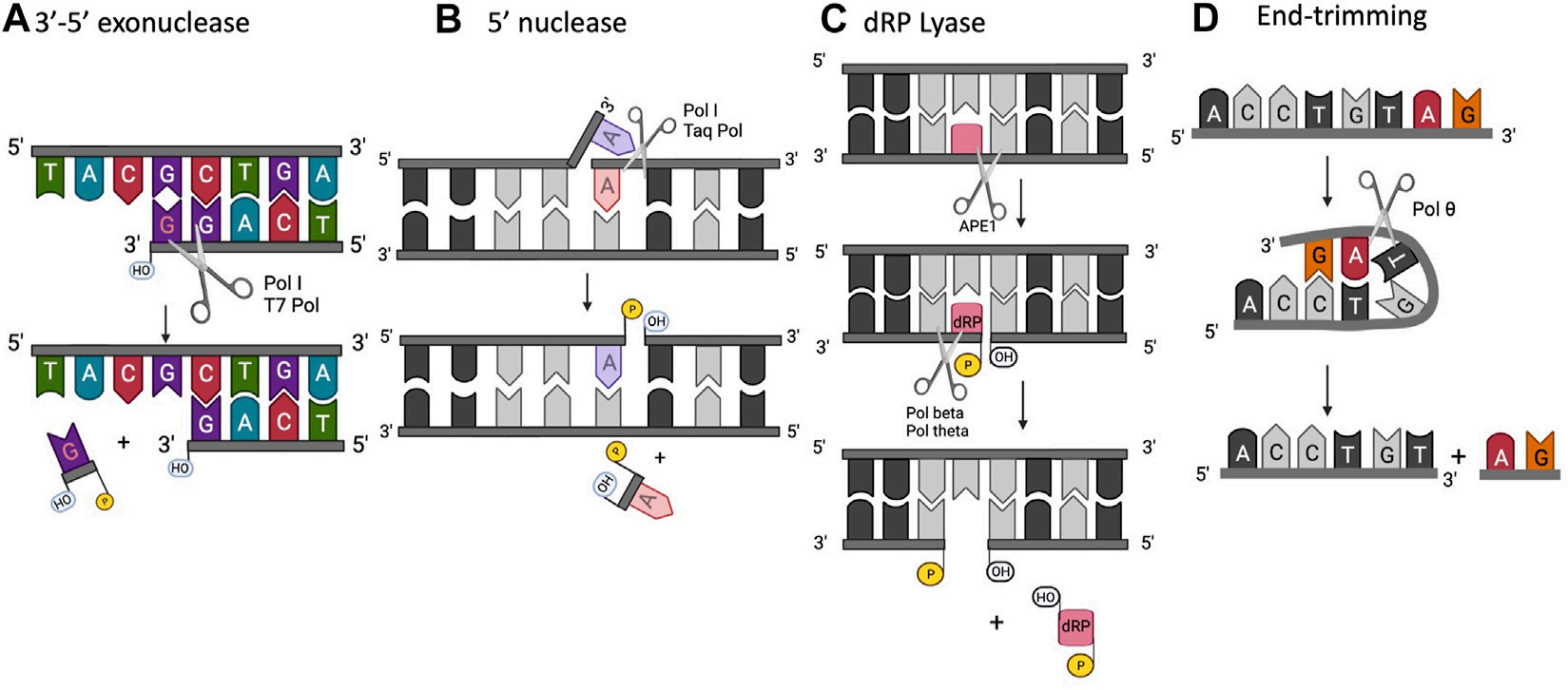
Extracurricular functions of DNA polymerases. **(A)** 3′-5′ exonuclease, **(B)** 5′ structure-specific nuclease, **(C)** dRP lyase, **(D)** end-trimming nuclease.

DNA polymerases have been intensively studied for decades because of their fundamental importance in DNA replication. Organisms throughout nature possess an array of polymerases encoded by their genomes, specialized for functions in DNA repair, recombination, and DNA damage tolerance. The canonical DNA polymerase reaction is the addition of a nucleotide, usually a deoxynucleoside triphosphate, to the 3′ end of a growing DNA chain, liberating pyrophosphate (Figure 1). The reverse reaction, pyrophosphorolysis, catalyzed by some DNA polymerases, is driven backwards by an excess of pyrophosphate. The purpose of this review is to highlight the existence of additional activities associated with DNA polymerases beyond canonical nucleotidyl transfer (Figure 2). We briefly summarize each activity, emphasizing recent results and unsolved issues.

### 3′–5′ Exonuclease Activity

All DNA polymerases share a common polymerase fold, which has been compared to a human right hand, composed of three subdomains: fingers, palm, and thumb (Steitz, 1999). In addition, the DNA polymerase toolbox can include a 3′–5′ exonuclease domain whose main role is to proofread new DNA synthesis to remove nucleotides that have been incorrectly incorporated. This reduces the error rate of DNA polymerases by one or more orders of magnitude during DNA replication (Joyce, 2013). In certain contexts, this 3′–5′ exonuclease activity is essential. Loss of editing activity results in increased incidence of cancer and pronounced genome instability (Shevelev and Hübscher, 2002; Shanbhag et al., 2018).

**FIGURE 3:**
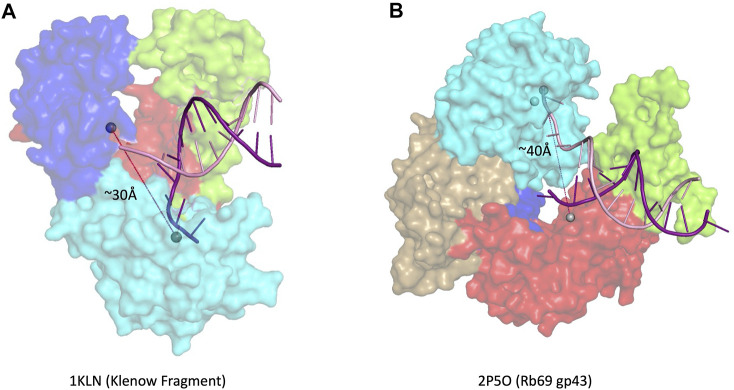
3′–5′ exonuclease. **(A)** Family- A polymerase Klenow fragment with DNA in the editing site (1KLN) (Beese et al., 1993), **(B)** Family-B polymerase RB69 gp43 with DNA in the editing site (2P5O) (Hogg et al., 2004). Subdomain color scheme same as Figure 1, with RB69 gp43 N-terminal domain in gold.

Multiple families of DNA polymerases harbor a 3′–5′ exonuclease activity: B-, C-, and D-family replicative polymerases (δ and ε in eukaryotes, Pol III in *E. coli,* PolD in euryarchaeota) and A-family repair polymerases, such as *E. coli* Pol I (Filée et al., 2002). Many A-family and some B-family polymerases have a conserved 3′–5′ exonuclease domain located in the N-terminal region of the larger polymerase domain (Khare and Eckert, 2002). In other B-family polymerases, the location of the exonuclease domain is found in a structurally distant region. The exonuclease active site in *E. coli* DNA polymerase I, an A-family polymerase, is ∼30 Å away from the polymerase active site (Figure 3A), whereas the distance between the two active sites is closer to ∼40 Å in B-family polymerases (Figure 3B) (Franklin et al., 2001; Hogg et al., 2004). Most B-family DNA polymerases have an extended β-hairpin loop that facilitates primer movement by holding the template strand in place as the primer switches into the exonuclease active site, a feature not found in A-family polymerases with 3′–5′ exonuclease activity (Hogg et al., 2007).

Although homology among DNA polymerases varies significantly, the 3′–5′ exonuclease active site itself is very conserved within A-family polymerases. There are three highly conserved amino acid regions containing critical residues that coordinate two divalent metal ions, ssDNA and deoxyribonucleoside monophosphates (dNMPs) (Bernad et al., 1989; Pinz and Bogenhagen, 2000). A key catalytic step required for 3′–5′ exonuclease activity is the chelation of two metal ions by active-site aspartates and glutamates (carboxylates) (Park et al., 1997). The Exo I, Exo II, and Exo III regions are essential for the exonuclease activity, as they contain the carboxylates that directly bind the metal ions required for catalysis (Bernad et al., 1989). Recent studies have identified other residues that help facilitate catalysis in the exonuclease active site. In bacteriophage ϕ29 DNA polymerase, tyrosine, threonine, and glutamine residues help coordinate the binding of DNA to the exonuclease active site. Specifically, Tyr 101 and Thr 189 assist in melting dsDNA to form ssDNA that can bind the active site (Rodriguez et al., 2019). Although *Thermus aquaticus* DNA polymerase (Taq) comprises an exonuclease domain, the enzyme lacks the three aspartates and one glutamate essential for catalysis and therefore has no proofreading activity (Kim et al., 1995); nevertheless exonuclease activity can be conferred to some extent by introducing the four carboxylates into the Taq sequence (Park et al., 1997).

To ensure replication fidelity the exonuclease activity operates in a delicate balance with the polymerase activity, with the exonuclease favored when a mismatch is inserted (Doublié and Zahn, 2014). The enzyme contributes to this regulation by monitoring the newly incorporated bases. The polymerase senses a mismatch by using strategically positioned protein residues that detect the minor groove N3 and exocyclic O2 positions of incorrect base pairs after incorporation, such as Arg 429 and Gln 615 in T7 DNA polymerase, and Tyr 567 and Lys 706 in RB69 gp43, respectively (Doublié et al., 1998; Franklin et al., 2001; Hogg et al., 2004). In Family B DNA polymerase ϵ, conserved Lys 967 and Arg 988 were found essential in regulating the switching of the DNA primer between the polymerase active site and exonuclease active site. Both residues lie between the palm and thumb domains and directly interact with the minor groove (Ganai et al., 2015). When a mismatch is present, the melting temperature of the bases at the 3′ terminus is lower favoring the formation of ssDNA ends. In fact, the binding of at least 3-4 bp ssDNA in the exonuclease active site is required to favor the cleavage reaction over the polymerase reaction, (Derbyshire et al., 1988; Beese et al., 1993). The functional separation of the two active sites also helps in the regulation of the exonuclease activity, to ensure it is used sparingly and only when mismatches are introduced. To achieve proper binding in the exonuclease active site, the enzyme must translocate backwards, leading to sufficient DNA unwinding to allow the primer terminus to leave the polymerase active site and bind the exonuclease active site (Beese et al., 1993). More recent FRET experiments mapped the trajectories recorded by a DNA primer translocating from the polymerase active site to the exonuclease active site of the Klenow fragment of DNA pol I. These data revealed that the enzyme can shuttle the primer between the two sites without dissociating from DNA (intramolecular transfer) (Lamichhane et al., 2013).

In some B-family polymerases, the two domains may be more structurally distant, complicating this active site switching step. To overcome this obstacle, some polymerases can bend the DNA in the active site to bring the exonuclease domain closer to the polymerase domain, facilitating the cleavage reaction. In the bacteriophage ϕ29 DNA pol, the primer terminus moves in one dimension from the polymerization site to the exonuclease site by a 40° rotation in the helix of the DNA (Khare and Eckert, 2002; Del Prado et al., 2018). In addition to the conserved catalytic aspartates in the exonuclease domain discussed above, a non-catalytic conserved aspartate with a regulatory role has recently been identified (Del Prado et al., 2018). In ϕ29 DNA pol, this residue, Asp 121, is located between the Exo II and Exo III motifs. This Asp facilitates the binding of ssDNA substrates at the 3′–5′ exonuclease active site, but, interestingly, it does not come into contact with the metal ions or the ssDNA. Asp 121 facilitates the melting of dsDNA to form the ssDNA primer required to catalyze the cleavage reaction. Similar invariant aspartates have also been identified in other enzymes, such as Asp 150 in *E. coli* RNase T (Zuo and Deutscher, 2002). In bacteriophage T7 DNA pol, mutating the predicted invariant Asp90 in the active site of the exonuclease domain completely ablated the exonuclease activity of the enzyme (Del Prado et al., 2018). The proximity of Asp 90 to multiple residues required for the exonuclease reaction suggested to the authors that this residue functions in a “Romanesque vault” structure, facilitating bending of the DNA to bring the primer into the correct orientation in the active site for cleavage (Del Prado et al., 2018).

Even further redundancies and regulators have been found that facilitate the switch between polymerase activity and exonuclease activity, which is essential for replication fidelity. The switch between polymerase active site and exonuclease active site is also controlled by the concentration of dNTP present in the active site (Beechem et al., 1998). Decreased occupancy of the polymerase active site by incoming dNTP shifts the balance towards exonuclease catalysis. More recently, it was shown that phosphorylation of yeast Pol ε during replication fork stalling can also shift the equilibrium. Checkpoint kinases mediate the phosphorylation of Ser 430 in *S. cerevisiae* pol2, preventing primer DNA switching to exonuclease sites. This post-translational modification regulates the DNA switching between the polymerase active site and the exonuclease site, limiting fork resection and subsequent collapse (Pellicanò et al., 2021).

Beyond proofreading, polymerase 3′–5′ exonuclease activity is important in promoting virus genetic recombination, as seen in Poxviruses. Vaccinia DNA Polymerase mutants lacking 3′–5′ exonucleolytic activity showed a dramatic reduction in recombination (Gammon and Evans, 2009). The authors propose a model in which the polymerase may use 3′–5′ exonuclease activity to rescue a double-strand break (DSB) by exposing sufficient homology in ssDNA to allow annealing at the break, repair by DNA polymerase and ligase, and subsequent resumption of DNA replication (Gammon and Evans, 2009).

### 5′-Nuclease Activity

**FIGURE 4:**
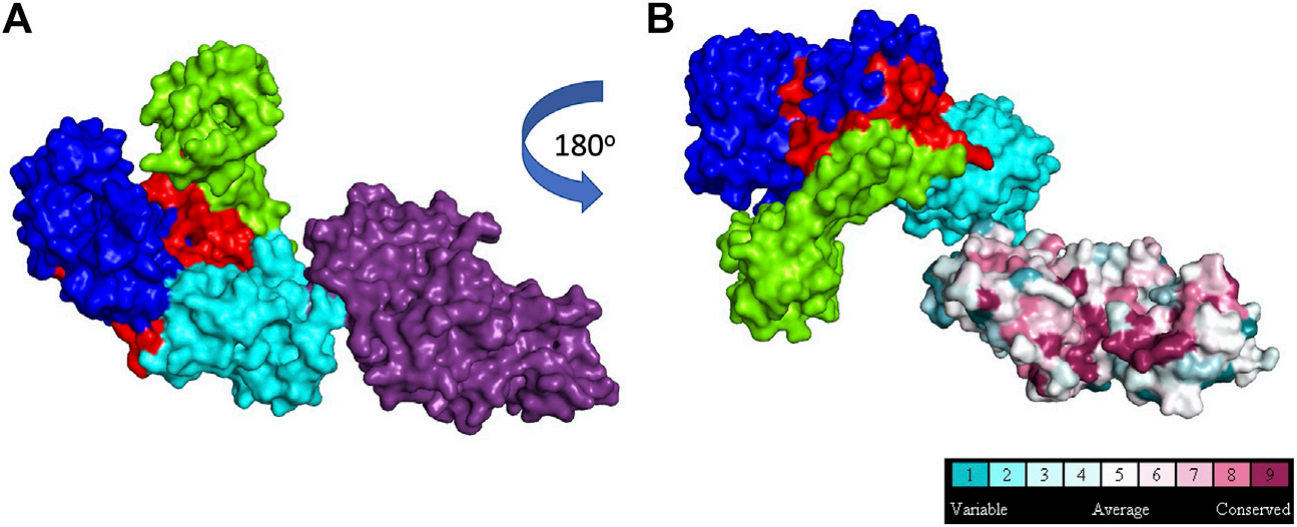
5′ structure specific nuclease. **(A)**: Taq polymerase with 5′ nuclease domain shown in purple, **(B)**: Taq polymerase rotated 180^o^ to show 5′ nuclease residue conservation across 150 homologs (1TAQ) (Kim et al., 1995). Subdomain color scheme same as Figure 1.

Some DNA polymerases, such as Taq, also possess a 5′- nuclease activity located in a separate domain (Figure 4) (Longley et al., 1990; Lyamichev et al., 1999). The 5′-nuclease activity was originally referred to as a 5′–3′ exonuclease activity, but it has since been established as a structure specific-cleavage of a 5′-ssDNA end joined to duplex DNA, a structure that is formed during lagging strand DNA synthesis (Joyce, 2013). Conserved residues in the 5’ nuclease domain (Gutman and Minton, 1993) share homology with the flap endonuclease (FEN1/XPG) family of proteins (Harrington and Lieber, 1994; Robins et al., 1994; Patel et al., 2001; Tsutakawa et al., 2011). In *E. coli* Pol I, the 5′-nuclease domain is tethered to the polymerase domain by an unstructured 16 amino acid peptide that is susceptible to proteolytic cleavage, allowing isolation of a Pol I “Klenow fragment” that lacks the 5′-nuclease domain (Klenow and Overgaard-Hansen, 1970). As such, the Pol I 5′-nuclease primarily operates during lagging strand synthesis to remove 5′ flaps formed when Pol I encounters Okazaki fragments during synthesis. The 5′-nuclease activity recognizes the 5′ flap and cuts between the first two bases, creating a nick on the DNA that is subsequently filled by DNA ligase (Ceska and Sayers, 1998; Xu et al., 2000).

DNA travels between the polymerase and 5′- nuclease domains through several conformational transitions (Joyce et al., 1992). The 5′-nuclease domain is flexible and adopts different positions within the Pol I-DNA complex to remove 5′ flaps (Ceska and Sayers, 1998). The polymerase and 5′ nuclease active sites compete for access to DNA ends, with dramatic shifts between the two active sites occurring during ongoing lagging strand synthesis. The 5’ nuclease activity also requires 2 divalent metal ions for cleavage. The selection of activity is likely intricately regulated by molecular “gates” in the active site, although details of this regulation are unknown (Joyce, 2013). However, the intricate gating mechanisms in FEN family proteins that help discern between different substrates can be used to glean insights into how 5′- nuclease is regulated in Pol I (Grasby et al., 2012; Pauszek et al., 2021).

### 5′-dRP Lyase Activity

Steps in base excision repair (BER) occur in a coordinated manner and enzymes are sequentially displaced to facilitate the next step in the pathway. During BER, a damaged base (for example, one modified by reactive oxygen species) is removed by a lesion-specific DNA glycosylase. This process leaves an apurinic/apyrimidinic (AP) site or abasic site that is recognized and cleaved by an AP endonuclease. This cleavage leaves a 5′-terminal deoxyribophosphate (dRP) residue that must be removed in concert with replacement of the base. In mammalian cells, Pol β is the main DNA polymerase involved in BER, and it is able to carry out both of these enzymatic steps. Pol β removes 5′- dRP and fills the gap (Matsumoto and Kim, 1995; Sobol et al., 1996; Allinson et al., 2001).

**FIGURE 5:**
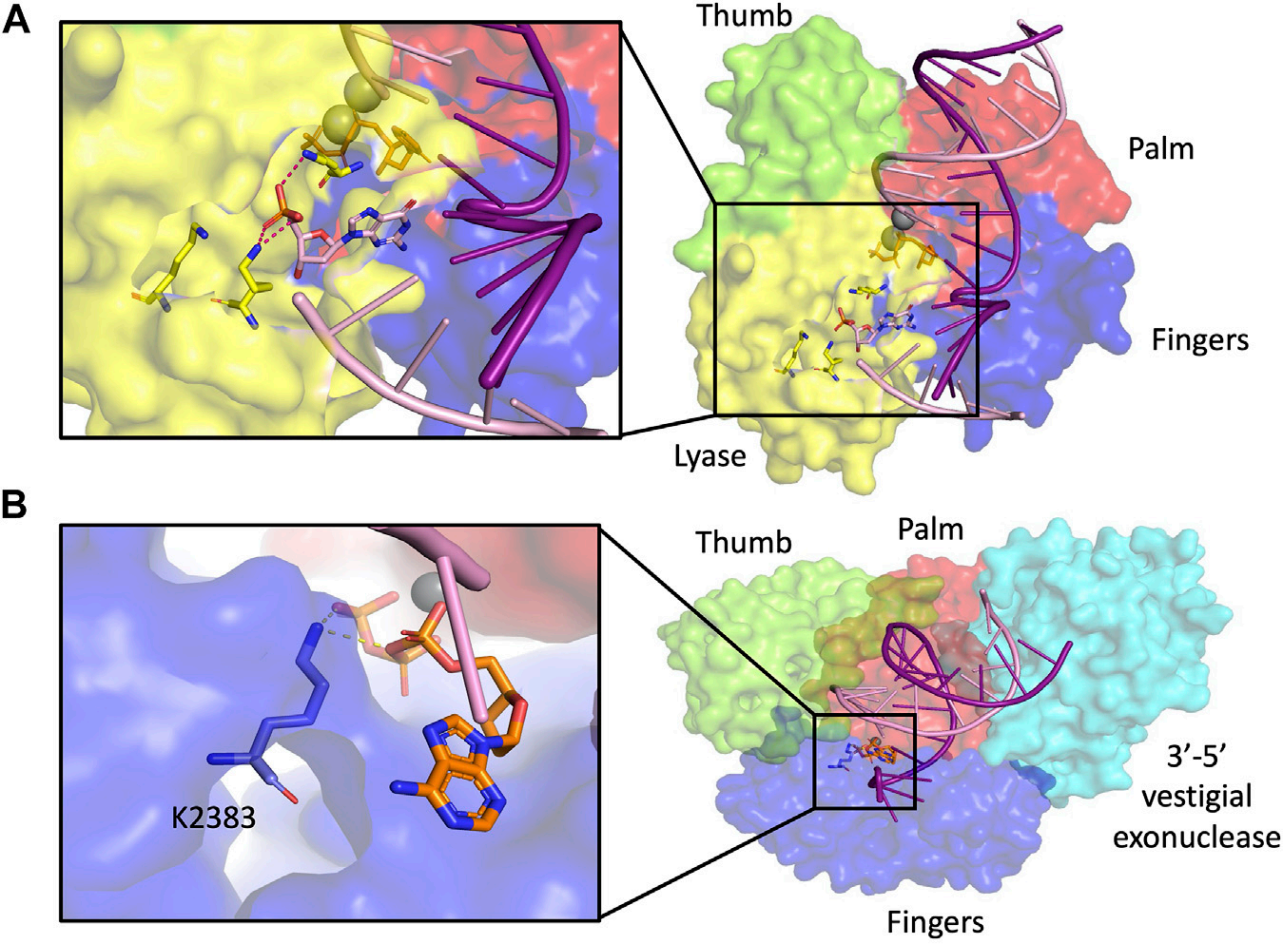
dRP lyase. **(A)** Pol β structure with dRP lyase residues highlighted in yellow in magnified inset (2FMS) (Batra et al., 2006), **(B)** Pol θ structure with dRP lyase residue highlighted in blue in magnified inset (4×0P) (Zahn et al., 2015). Subdomain color scheme same as Figure 1.

The 5′-dRP lyase activity is contained within an 8 kDa N-terminal domain of Pol β (Prasad et al., 1994; Prasad et al., 1998) (Figure 5A). A metal ion is required for release of the 5′dRP from double-stranded DNA allowing the gap in the DNA to be filled and subsequently ligated (Matsumoto and Kim, 1995).

A nick adjacent to a 5′-dRP residue is a small target, and Pol β must bind non-specifically along the DNA as it searches for the right substrate to cleave. PARP1 has a high affinity for this substrate and may help recruit pol β (Khodyreva et al., 2010; Prasad et al., 2014). Recently, it was found that three lysines in the lyase active site of Pol β facilitate the search for the 5′- flap substrate on the dsDNA and destabilize non-specific DNA binding. Mutating these three lysine residues to alanines in the lyase active site of Pol β (Lys 35, Lys 68, and Lys 72), increased the binding affinity of Pol β for nonspecific DNA, indicating a role of the 5′dRP lyase in the specificity of lesion recognition (Howard et al., 2020). A tumor-associated variant in the N-terminal 8 kDa domain of Pol β retains polymerase activity but is dRP lyase deficient (Dalal et al., 2008). DNA methylating agents such as methyl methane sulfonate (MMS) create base lesions that are removed by BER. Fibroblasts of Pol β null mice are hypersensitive to MMS. This sensitivity can be rescued by complementation with the 5′dRP lyase activity of Pol β, without requiring the DNA polymerase activity (Sobol et al., 2000). In this case, another DNA polymerase such as pol δ accomplishes gap filling (Klungland and Lindahl, 1997; Fortini et al., 1998). Furthermore, Pol β 5′dRP lyase activity is suggested to be important in preventing trinucleotide repeat instability. Pol β defective in 5′-dRP lyase activity can be tightly bound to a repair site, forcing slippage that can result in deletions of trinucleotides (Lai et al., 2018).

Other polymerases also carry out 5′-dRP lyase activity. Y-Family pol iota (Pol ι) possesses 5′-dRP lyase activity and can alleviate BER deficiency *in vitro* (Bebenek et al., 2001; Prasad et al., 2003). Loss of Pol ι sensitizes the cells to treatment with oxidative damaging agents (Petta et al., 2008). Pol ι accumulates in sites of oxidative DNA damage and associates with BER modulator XRCC1 to facilitate repair. The recruitment of Pol ι to sites of DNA damage in human cells requires the 5-dRP lyase domain of the enzyme. It is not clear whether the dRPase of Pol ι is functionally relevant to its operation in a pathway to counteract DNA replication stress (Wang et al., 2020), involving p53-dependent reactivation of DNA replication forks (Hampp et al., 2016; Biber et al., 2020).

The A-Family polymerase Pol θ also possesses 5′-dRP lyase activity within its polymerase domain. *In vitro*, Pol θ can participate in BER reactions (Prasad et al., 2009). Lys 2383, a residue critical for AP lyase, is also important for the DNA polymerase activity of Pol θ (Figure 5B) (Laverty et al., 2018). The 5′-dRP lyase activity of Pol θ can also operate in structurally clustered lesions (Laverty and Greenberg, 2018). In these clustered regions, Pol β is 15–20 fold slower at excising the 5′-dRP lyase compared to Pol θ, which suggests that the two polymerases may have evolved to take care of a larger variety of substrates, with Pol β being favored for excising lesions with opposite polarity than those excised by Pol θ (Laverty and Greenberg, 2018). BER is also important for repair of oxidative lesions in mitochondrial DNA. Mitochondrial DNA polymerase y (Pol γ) also possesses 5′-dRP lyase activity, although with a much slower release of the dRP group from the enzyme compared to Pol β. In fact, the 5′-dRP lyase activity in A-family polymerases such as Pol γ, Pol θ, and Pol I is significantly slower than in Pol β, which suggests that this function may not be sufficient to complete repair of abundant AP sites (Pinz and Bogenhagen, 2000).

### 3′-End-Trimming and Single-Strand Extension

**FIGURE 6:**
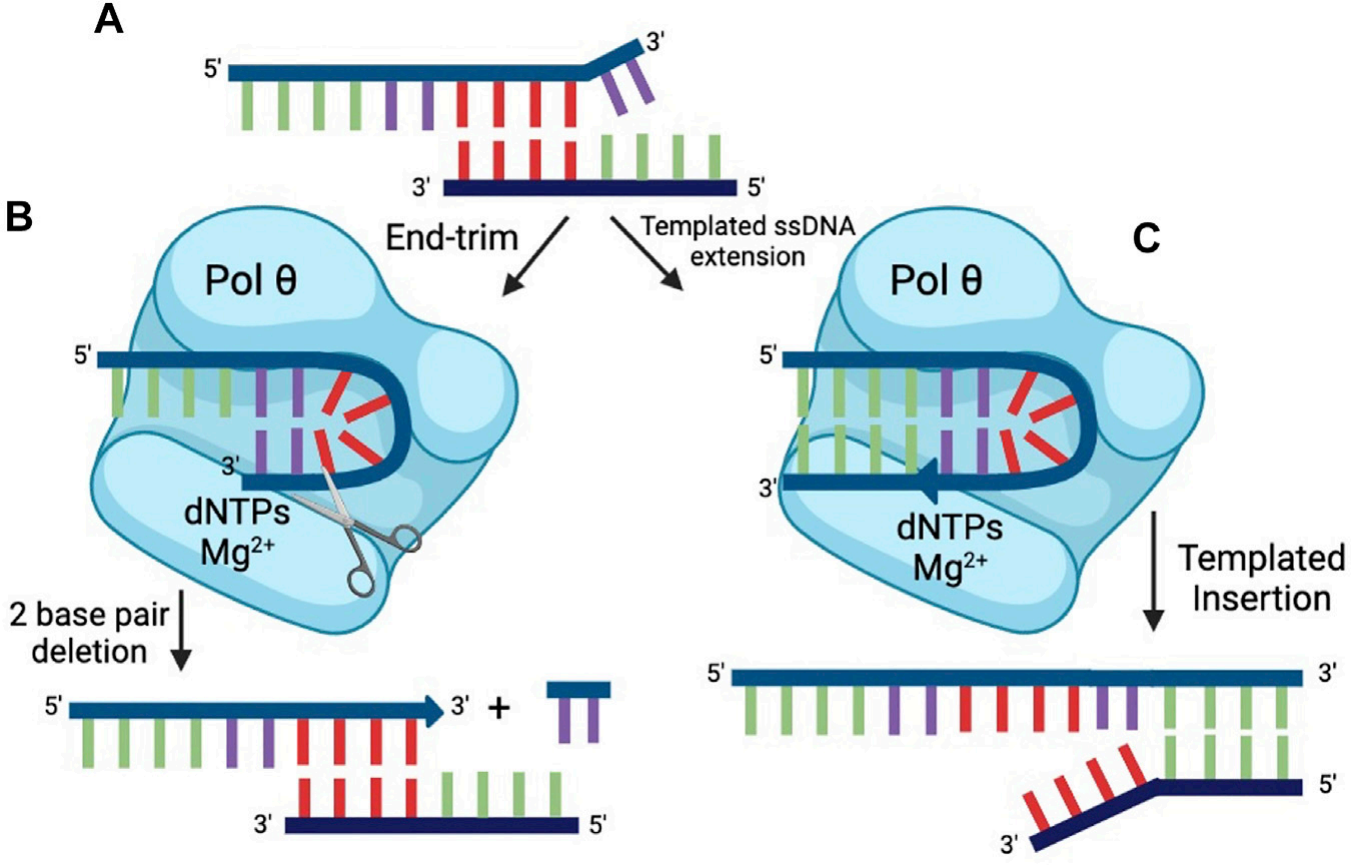
End-trimming. **(A)** The top strand of DNA cannot serve as a primer for successful repair because the microhomology (red) is internal. Two terminal 3′ nt (violet) are unpaired. Pol θ can process ssDNA ends by two modes: **(B)** Transient self-pairing of a 3′ end within pol θ (violet nt) can lead to end-trimming catalysis, resulting in removal of 2 nt (in this case). This allows the internal microhomology (red) to engage in productive priming. **(C)** Transient self-pairing of a 3′ end within pol θ (violet nt) can alternatively result in limited synthesis. In this case, 4 nt (green) that are complementary to an internal microhomology on the bottom strand of DNA are synthesized. The result of this process is a short, templated insertion.

A DNA end-trimming activity, distinct from the nuclease activities discussed above, was identified in human Pol θ (Zahn et al., 2021). The rapid end-trimming activity acts on single-stranded DNA when placed in a transient and appropriate self-pairing conformation within pol θ (Figure 6). This end-trimming activity is different from 3′ to 5′ exonuclease activity. Although Pol θ contains a proofreading domain, it lacks two of the conserved amino acids necessary for 3′–5′ exonuclease activity. Further, a short loop in the exonuclease-like domain of pol θ would impede access of DNA to the exonuclease active site (Zahn et al., 2015).

This end-trimming activity shares many of the dependencies and catalytic residues that are required for the DNA polymerase reaction. For example, end-trimming is dependent on the presence of divalent cation (Mg^2+^ or Mn^2+^), on catalytic residues including invariant aspartate Asp 2330 that coordinates Mg^2+^ in the active site, and on the addition of dNTPs (Zahn et al., 2021). Catalysis of both extension and nuclease activity from the same active site is plausible in principle because the two-metal ion active sites of nucleotidyl transfer enzymes share close similarities with the active sites of many nucleases (Yang et al., 2006).

Experimentally, dNTP-dependent end-trimming activity is observed with some, but not all single-stranded oligonucleotides. For those substrates that are end-trimmed, a unifying feature is that the 3′ terminus is capable of potential self-pairing with a short sequence within the oligonucleotide, suggesting that a hairpin-type configuration is transiently formed within the active site of pol θ (Figure 6B). On the most-studied substrate, 2 nt are cleaved from the 3’ terminus in an endonucleolytic reaction; on some substrates 1 nt is removed; on others, possibly more than 2 nt may be end-trimmed. The end-trimming configuration appears to be unimolecular, because all substrate is quickly consumed. Further, the proposed end-trimming configuration explains another activity of pol θ, the ability to extend single-stranded DNA substrate.

Pol θ DNA polymerase can extend some single-stranded DNA molecules, an activity that is not shared by other known A-family polymerases (Hogg et al., 2012). In the presence of Mg^2+^, the products observed are both primed and largely templated by the same oligonucleotide (Figure 6C) (Hogg et al., 2012; Yousefzadeh et al., 2014). It was suggested that the single-stranded extension activity of Pol θ was the result of non-templated terminal transferase activity (Kent et al., 2016). However, Pol θ is not a terminal transferase. Experiments in the study suggesting terminal transferase activity were conducted using Mn^2+^. This metal leads to promiscuous synthesis and a high rate of mismatch incorporation, and it is known that Pol θ can extend readily from a 3′-terminal mismatch (Seki and Wood, 2008; He and Yang, 2018). Like other A family polymerases, Pol θ can add an additional dATP to a blunt DNA end, but otherwise shows predictable template requirements in a first round of DNA synthesis (He and Yang, 2018).

DNA polymerase θ is the defining enzyme in a double strand break repair pathway, termed theta-mediated end-joining (TMEJ). During TMEJ, Pol θ engages single stranded 3′ ends by microhomology. It thus makes sense that Pol θ recognizes and processes ssDNA in several ways. Pol θ apparently manipulates the 3′ end of the ssDNA in the active site, forming a transitory hairpin that serves as the intermediate for either cleavage or extension of the 3′-end (Zahn et al., 2021). The factors that dictate the choice between these two activities are being investigated, as well as how processed single-strands are then advanced to a repair stage that uses pairing at microhomologies and primed extension on double-stranded DNA. Statistically, microhomologies will rarely be found at the ends of a double-strand break but will more often occur internal to the break (Figure 6A). The two steps of processing of DNA ends and templated extension appear to largely explain the mutational “signature” of TMEJ, involving short sequence insertions and deletions at the site of repair (Schimmel et al., 2019; Carvajal-Garcia et al., 2020; Hwang et al., 2020).

Endonucleolytic cleavage of a polynucleotide chain is also a feature of RNA polymerases, which edit transcripts by cleaving off 1 or 2 nucleotides from the 3′ end (Izban and Luse, 1992; Reines, 1992; Borukhov et al., 1993). This editing reactions are conducted in a Mg^2+^ - dependent manner from the RNA polymerase active site. The regulation of extension vs. cleavage is enhanced by protein cofactors that participate directly in reorganizing the amino acids in the catalytic site, referred to as “active center tuning” (Sosunova et al., 2013). For example, the endonuclease activity of *E. coli* RNA polymerase is stimulated by Gre proteins, and eukaryotic RNA pol II by TFIIS. Stimulated by Gre, RNA polymerase clips off the two terminal nt of a synthetic RNA fragment (Sosunov et al., 2003; Sosunova et al., 2013). In contrast to the pol θ end-trimming reaction, RNA polymerase editing does not seem to be directly dNTP- or NTP-dependent, although it can be modulated by nucleotide concentration. Other nucleotidyl transferases may also engage in a form of end-trimming or editing. For example, telomerase, including highly purified human telomerase, can shorten products as well as extend them (Collins and Greider, 1993; Rubtsova et al., 2012; Fouquerel et al., 2016).

### Activity on RNA

Some DNA polymerases perform nucleotidyl transferase reactions that do not involve the usual DNA substrates or primers, and are briefly mentioned here to further illustrate the enzymatic versatility of DNA polymerases.

### Reverse Transcriptase

Reverse transcriptases are RNA-dependent DNA polymerases, which use an RNA template to synthesize DNA. Some DNA-dependent DNA polymerases have been reported to have reverse transcriptase activity. Two bacterial A-family polymerases, *Bst* DNA polymerase and the Klenow fragment of *E. coli* Pol I, were successfully used to reverse transcribe RNA *in vitro* (Shi et al., 2015). Taq DNA polymerase can also engage in this activity, after mutating a single aspartate into asparagine (Barnes et al., 2021). Human pol η can use either a DNA duplex or and RNA/DNA mixed duplex during synthesis (Franklin et al., 2004; Su et al., 2017), and was shown to function as a reverse transcriptase in a cellular environment (Su et al., 2019). Pol θ was recently reported to also have a reverse transcriptase activity, although additional studies are warranted to establish the physiological relevance of this finding (Chandramouly et al., 2021).

### Primase-Polymerase

Some DNA polymerases, for example eukaryotic DNA pol α, have a separate primase subunit that allows RNA priming for DNA replication. PrimPol is the only known eukaryotic DNA polymerase with intrinsic priming activity. Both primase and DNA synthesis are mediated by the same active site. Recently it was found that a priming activity of DNA polymerase PrimPol is used for replication restart after DNA damage (Garcia-Gomez et al., 2013; Mouron et al., 2013). PrimPol is primarily a TLS polymerase which is found in both the nucleus and the mitochondria, and is capable of bypassing 7,8-dihydro-8-oxoguanine (oxoG) and cyclobutane pyrimidine dimers (CPD) (Mouron et al., 2013; Rechkoblit et al., 2021). PrimPol can synthesize short DNA primers which other polymerases, like mitochondrial polymerase γ, can use to restart replication promoting overall mitochondrial genome maintenance. Recent studies have found that PrimPol repriming activity is used by stalled replication forks to skip DNA lesions and restart replication, leaving behind ssDNA gaps that can be filled by other repair polymerases (Quinet et al., 2020; Tirman et al., 2021).

## Conclusion

DNA polymerases catalyze the nucleotidyl transfer reaction to generate a DNA polymer. In practice, however, this class of enzymes is able to catalyze a host of other DNA processing reactions that make DNA polymerases a multi-tool of genomic integrity. Redundancy in the activities of DNA polymerases is critical for replication and maintenance of the cell. If a DNA polymerase is unable to perform its function, other DNA polymerases are poised to intervene and assume the role of replication and repair typically filled by the compromised enzyme. These redundancies also facilitate the sheer amount of DNA replication and repair that must continuously occur in the cell throughout its life. It must be noted that the existence of redundant polymerase roles makes targeting DNA polymerases in cancer a challenge due to the ability of the cell to compensate for the loss by employing a different polymerase.

By using a DNA polymerase to execute more than one aspect of DNA processing, the cell can benefit energetically. Requiring a separate enzyme for each step would necessitate a greater energetic commitment from the cell to synthesize and recruit these enzymes than employing an existing polymerase. Furthermore, repurposing the active site of a DNA polymerase can facilitate spatial organization of enzymatic activities. By utilizing a common active site for multiple reactions, the polymerase becomes a hub where each activity may be performed sequentially. This arrangement is advantageous to the cell as a polymerase can process DNA in multiple ways before diffusing from the DNA, further minimizing the amount of energy required to attain appropriately processed DNA.

While DNA polymerases have been investigated for close to 60 years, we continue to discover noncanonical polymerase activities. From the addition of an enzymatic domain to exploiting an existing active site, DNA polymerases act like Swiss army knives of DNA processing. We can look forward to discovering what other functions these DNA multi-tools utilize to tackle DNA processing errors. Conveniently these multiple functions present additional opportunities to design small molecule inhibitors against these multitasking enzymes.
